# Minocycline differentially modulates human spatial memory systems

**DOI:** 10.1038/s41386-020-00811-8

**Published:** 2020-08-24

**Authors:** Sam C. Berens, Chris M. Bird, Neil A. Harrison

**Affiliations:** 1 School of Psychology, University of Sussex, Falmer, BN1 9QH UK; 2 Cardiff University Brain Research Imaging Centre, Cardiff University, Cardiff, CF24 4HQ UK; 3 Department of Neuroscience, Brighton and Sussex Medical School, University of Sussex, Brighton, BN1 9RR UK

**Keywords:** Microglia, Hippocampus

## Abstract

Microglia play a critical role in many processes fundamental to learning and memory in health and are implicated in Alzheimer’s pathogenesis. Minocycline, a centrally-penetrant tetracycline antibiotic, inhibits microglial activation and enhances long-term potentiation, synaptic plasticity, neurogenesis and hippocampal-dependent spatial memory in rodents, leading to clinical trials in human neurodegenerative diseases. However, the effects of minocycline on human memory have not previously been investigated. Utilising a double-blind, randomised crossover study design, we recruited 20 healthy male participants (mean 24.6 ± 5.0 years) who were each tested in two experimental sessions: once after 3 days of Minocycline 150 mg (twice daily), and once 3 days of placebo (identical administration). During each session, all completed an fMRI task designed to tap boundary- and landmark-based navigation (thought to rely on hippocampal and striatal learning mechanisms respectively). Given the rodent literature, we hypothesised that minocycline would selectively modulate hippocampal learning. In line with this, minocycline biased use of boundary- compared to landmark-based information (*t*_980_ = 3.140, *p* = 0.002). However, though this marginally improved performance for boundary-based objects (*t*_980_ = 1.972, *p* = 0.049), it was outweighed by impaired landmark-based navigation (*t*_980_ = 6.374, *p* < 0.001) resulting in an overall performance decrease (*t*_980_ = 3.295, *p* = 0.001). Furthermore, against expectations, minocycline significantly reduced activity during memory encoding in the right caudate (*t*_977_ = 2.992, *p* = 0.003) and five other cortical regions, with no significant effect in the hippocampus. In summary, minocycline impaired human spatial memory performance, likely through disruption of striatal processing resulting in greater biasing towards reliance on boundary-based navigation.

## Introduction

Progress in psychoneuroimmunology over the last 2–3 decades has revealed that medial temporal lobe (MTL) memory systems are heavily dependent on immunoregulatory pathways. Furthermore, interactions between peripheral and central (brain) immune systems can impair MTL-dependent memory [1, 2], and are implicated in the aetiology of many neurodegenerative disorders including Alzheimer’s disease [3]. Microglia (resident brain innate immune cells) play a key role in many processes fundamental to memory function in health including long-term potentiation (LTP) and neural plasticity, homoeostatic synaptic scaling, and neurogenesis [4, 5]. They are also strongly implicated in the aetiology and progression of Alzheimer’s and other neurodegenerative diseases [6] making them an attractive target for novel disease-modifying immunotherapies.

Medial temporal lobe structures appear to be particularly sensitive to the effects of inflammation. This is perhaps due to their relatively high density of microglia, the cytokine interleukin-1β (IL-1β), its receptor, and its naturally occurring antagonist (IL-1ra) [7, 8]. Within the hippocampus, IL-1β gene expression increases during, or shortly after learning [9] and appears critical to the maintenance of long-term potentiation (LTP) [10]. Similarly, microglial release of tumour necrosis factor (TNF-α) is implicated in the regulation of hippocampal synaptic scaling [11]. Microglia also play a central role in synaptic pruning and plasticity [12] and are the likely source of TNF, which is both necessary and sufficient for homoeostatic synaptic scaling [13]. Under quiescent conditions, microglia support adult neurogenesis within the hippocampus [14], though their role in human striatal neurogenesis [15] is yet to be investigated. Within the hippocampus, microglia have also been implicated in the beneficial effects of environmental enrichment on neurogenesis [16]. In health, these actions assist in the remodelling of neural circuits to promote learning and memory [4].

In contrast to their role in healthy cognition, activation of microglia from resting to pro-inflammatory states is linked to impaired memory, suppression of hippocampal neurogenesis [17, 18] and the pathophysiology of Alzheimer’s and other neurodegenerative diseases [6]. They have also been implicated in obesity-associated cognitive impairments [19]. Minocycline, a centrally penetrant tetracycline antibiotic, has been shown to inhibit microglial activation and reverse inflammation-associated suppression of neurogenesis via mechanisms that appear distinct from its antimicrobial action [17]. In rodent Alzheimer models, minocycline attenuates neuronal cell death and improves cognitive impairment, as measured by hippocampus-dependent memory tasks [20]. Moreover, in healthy aged animals, it improves hippocampus-dependent spatial memory (water maze acquisition) [21]. Nevertheless, in both of these studies, minocycline had no beneficial effects on cognition in the healthy adult control animals, suggesting benefits on memory performance might be apparent only in animals with a compromised hippocampal system. In terms of its actions at the cellular level, minocycline reduces total microglia cell counts [21], enhances hippocampal (CA1) LTP and augments dendritic spine density [22] in healthy aged animals. In addition, in healthy (non-aged) animals it is associated with increased hippocampal neurogenesis [21].

Together, these findings support the benefits of minocycline in disease and have motivated a number of human clinical trials. However, it remains unclear whether minocycline confers beneficial or deleterious effects in healthy animals. Furthermore, studies of the therapeutic effects of minocycline across a range of human disorders including HIV-associated cognitive impairment [23], Schizophrenia [24] and mild Alzheimer’s disease [25] have been disappointing. In Motor Neuron Disease, minocycline has even been associated with accelerated functional decline [26].

Despite its use in numerous preclinical rodent studies and human clinical trials, the effects of minocycline on healthy human memory have yet to be investigated. To address this, we recruited 20 healthy male participants and tested them during fMRI on a spatial memory task designed to tap boundary- and landmark-based navigation (thought to depend on hippocampal and striatal learning mechanisms respectively). Each participant was tested in two experimental sessions: once after 3 days of Minocycline 150 mg (twice daily), and once 3 days of twice daily placebo utilising a double-blind, randomised crossover study design. Based on the rodent literature, we predicted minocycline would selectively modulate hippocampal versus striatal-based learning mechanisms.

## Materials and methods

### Participants

Twenty healthy, male right-handed non-smokers were recruited from the University of Sussex (UK) via online advertisement. All reported no history of neurological, psychiatric or immunological disorder, were medication-free and had either normal or corrected-to-normal vision. Females were excluded owing to the teratogenic potential of minocycline. Two participants were subsequently excluded due to technical difficulties with the virtual navigation task and data from 18 participants are reported (mean age: 24.55 years, SD: 5.03). The study was approved by the Brighton and Sussex Medical School Research Governance and Ethics Committee. All participants provided written informed consent and were compensated for their time.

### Study design

The study utilised a cross-over repeated measures design in which all participants were tested twice; once following minocycline, and once placebo. Minocycline (150 mg) or visually identical placebo was administered twice daily for 3 days prior to each study session. The order of minocycline vs placebo administration was counterbalanced across participants and the two study sessions were separated by at least 14 days.

### Spatial memory task

**Fig. 1:**
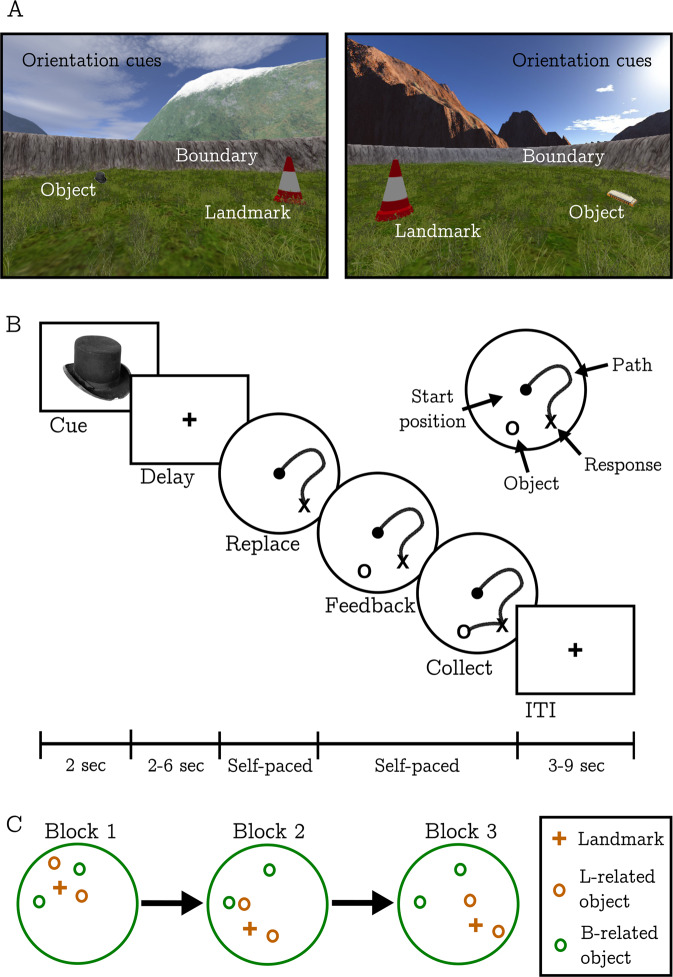
Virtual navigation task. **a** Examples of the two virtual environments. **b** Schematic illustration of the task procedure. **c** Examples of how the landmark, boundary and their associated objects moved relative to each other over the course of three blocks in order to train landmark vs boundary-based navigation. Within each block, there were four learning trials per object.

During fMRI, participants performed a modified version of a task designed to simultaneously tap two interacting learning systems that support spatial navigation [26]. This task has previously been used to show that these systems, which encode location with respect to environmental boundaries or an environment landmark, are differentially reliant on the hippocampus and dorsal striatum respectively [27]. The virtual environment was constructed in Unreal Engine 2 (Epic Games) and consisted of a circular grassy arena surrounded by a steep bank (boundary) and a non-centrally located traffic cone (landmark). Background mountains, clouds, and the sun projected at infinity were used to provide orientation cues without allowing location to be inferred by proximity or parallax. The boundary and landmark were both rotationally symmetric to avoid providing orientation information (Fig. 1a).

Participants’ goal was to learn the spatial location of four everyday objects placed in the arena. At the start of the task, participants entered the virtual arena with one object already in place. After identifying its location, they collected the object by walking over it. This was then repeated for each of the four objects. Participants then used trial-and-error learning to progressively improve how accurately they could relocate each object. This phase of the task consisted of three consecutive blocks, with each block containing 16 separate trials (four per object). Each trial started with an image of one of the objects presented for 2 s against a grey background (CUE). After a further 2–6 s delay participants were returned to the virtual environment at a random location without the object present (initial heading directions and start positions were randomly sampled from a uniform distribution, constrained to an area of two virtual metres in the centre of the arena). They were then required to move to where they believed the cued object was located and press a button (REPLACE). Immediately afterwards the true location of the object was revealed (FEEDBACK), and participants re-collected the object by walking over it. This final FEEDBACK phase allowed participants to progressive refine their memory regarding the true location of that object (Fig. 1b). Different objects, backdrops, and trained locations were used for each of the two separate testing sessions. Furthermore, object assignments to each navigational condition, the order of landmark/boundary shifts (see below), the distal backdrops, and the trained locations themselves were all counterbalanced across sessions.

To experimentally manipulate the use of the two (boundary vs landmark) navigational strategies two objects were assigned to a ‘boundary-related’ condition (B-RELATED), and two a ‘landmark-related’ (L-RELATED) condition. The relative position of the landmark and the boundary shifted twice during the task (at the start of blocks 2 and 3; not explicit in task instructions to mitigate potential demand characteristics). Note: these shifts should be properly understood as relative changes in landmark/boundary position since these cues were the only objects that provided proximity information in the environment. Following each shift, B-RELATED objects maintained a fixed position with respect to the boundary and L-RELATED objects a fixed position relative to the landmark (Fig. 1c). Consequently, during the FEEDBACK phase of blocks 2 and 3, the boundary and the landmark became differentially informative with respect to learning the locations of B- and L-RELATED objects. Of note, the landmark/boundary shifts were not restricted to simple rotations. This made it more likely that each object location was learned relative to its associated navigation cue since proximity and angular distance were both relevant. This task was identical to that used by Doeller et al. [27] with the exception that the original study involved four blocks rather than the three used here.

### Behavioural data analysis

Location estimates during the REPLACE phase were used to calculate drop-errors (in virtual metres) reflecting the absolute distance between estimated and true object locations during each trial. Drop-errors for each participant and trial were analysed using a generalised linear mixed-effects model (GLMM) with four fixed-effect predictors and their interactions: (1) drug (minocycline vs placebo), (2) navigational condition (landmark- vs boundary-related objects), (3) experimental block (block 2 vs 3), and (4) trial number (i.e. trials 1 to 4). Of note: As the distinction between landmark- and boundary-related objects was not made apparent until the start of block 2, data acquired prior to this point were not included in the analysis. Random-effect predictors were used to account for statistical dependencies between observations from the same participant and object. Drop errors were all non-negative and distributed with a large positive skew that closely approximated an Inverse-Gaussian distribution. Thus, the GLMM used an Inverse-Gaussian to parameterise the distribution of drop-errors with a log link-function to constrain predicted scores between 0 and positive infinity.

Following Doeller et al. [27], we also generated a metric (*LB-influence*) to quantify the relative influence of each navigational cue (landmark vs boundary) on REPLACE performance. We first calculated the distance between each replaced location, and the location of that object as predicted by either the boundary (dB) or the landmark (dL). LB-influence was then calculated as the ratio of dL to the sum of dB and dL i.e. dL ÷ (dB + dL). This metric varies between 0 and 1 with low values indicating greater reliance on the landmark, and higher values (closer to 1) greater reliance on the boundary. As an example, for an L-related object, dL is simply the drop-error and *dB* the distance between the replaced location and the boundary-related location that would have been correct prior to the most recent landmark shift. We again used a GLMM to estimate how LB-influence was modulated as a function of drug, navigational condition, block, and trial. This model had the same fixed- and random-effects structure as that used in the drop-error analysis. However, given that LB-influence scores are bounded between 0 and 1, the model used a logit link function to constrain predicted scores to this interval.

Based on the results of prior studies, we predicted that drop-errors would gradually decrease for all objects in each block. Similarly, we predicted that LB-influence scores would gradually diverge from ~0.5 (equal landmark- and boundary-influence) towards 0 or 1 for L-, and B-related objects, respectively. We therefore performed a linear contrast across the parameter estimates from trails 1 to 4 to test the main effects and interactions related to trial-by-trial differences.

### Effect of participant BMI

Obesity has been associated with deleterious effects on the CNS including accelerated age-related cognitive decline and medial temporal lobe (particularly hippocampal) atrophy in humans [19]. This has been linked to increased microglial activation and the release of IL-1β in rodents [19]. Given this, we performed a secondary analysis to explore possible interactions between body mass index (BMI) and effects of minocycline on behavioural performance using additional GLMMs of drop error and LB-influence statistics that included participant BMI as a continuous covariate (range: 17.57–31.40, mean centred). BMI was permitted to interact with the effects of drug, navigational condition, and their interaction to enable us to test for differential effects of minocycline in participants with different BMIs (for landmark vs boundary objects separately). However, to limit the number of fixed effects coefficients, we did not permit BMI to interact with experimental block or trial number. These interactions were found not to significantly interact with minocycline (see results), and the additional model complexity was not warranted based on model fit statistics (Bayesian Information Criterion) [28].

### MRI analyses

Details of the imaging protocol and pre-processing steps are provided in the Supplementary Methods. First-level fMRI models included individual boxcar regressors for the CUE, REPLACE, and FEEDBACK phases of each trial, an intercept term, and 12 motion parameters (realignment parameters and their first derivatives). First-level beta coefficient images from each participant and session were then used in two second-level linear mixed-effects models (LMM) to model BOLD activations during memory retrieval (REPLACE), and memory encoding (FEEDBACK). As previous work has demonstrated that the hippocampus and striatum differentially contribute to boundary- and landmark-based navigation [27, 29] we focused these analyses on four regions of interest (ROIs): the left and right hippocampus, and the left and right caudate nuclei (defined using the AAL atlas; shown in Supplementary Fig. S1) [30]. Specifically, average BOLD activity during each REPLACE/FEEDBACK trial was extracted for each ROI. To supplement our main analyses, we also examined BOLD effects outside our four ROIs using LMMs of the REPLACE and FEEDBACK data in 400 non-overlapping brain parcels (derived from a whole-brain parcellation by Schaefer et al. [31]).

The LMMs for memory retrieval (REPLACE) included four predictors of interest: (1) drug (minocycline vs placebo), (2) navigation condition (landmark- vs boundary-related objects), (3) LB-influence, (4) participant BMI, and all possible interactions between them (note: the inclusion of each model term was informed by the results of our behavioural analysis, as below). As LB-influence is believed to represent the relative contribution of landmark and boundary cues to navigation performance, we predicted this would correlate with BOLD activity during the replace phase. The LMM for memory encoding (FEEDBACK) also included four predictors: (1) drug (minocycline vs placebo), (2) navigation condition (landmark- vs boundary-related objects), (3) drop-error, and (4) participant BMI, and all possible interactions between them. Here, we assumed that drop-error statistics directly corresponded to prediction error during feedback, and in turn, reflect the degree of memory encoding [32].

As for the behavioural analyses, data acquired prior to trial 2 of block 2 were not included in either LMM of the fMRI data since the distinction between landmark- and boundary-related objects was not made apparent until this point. LB-influence and drop-error were logit- and log-transformed respectively before being *z*-scored to ensure that the sampling distribution of model parameters was approximately normal. In addition, by removing all bounds on the predictor values, these transforms allowed any changes in LB-influence and drop-error to be associated with meaningful differences in BOLD activity. Trial number was not included as a predictor in either LMM as behavioural results revealed that this was largely co-linear with both LB-influence and drop-error (see ‘Results’). Both LMMs also included random intercepts and slopes for each fixed effect factor. These accounted for statistical dependencies related to observations from the same participants, task blocks and objects.

## Results

### Behavioural task

**Fig. 2:**
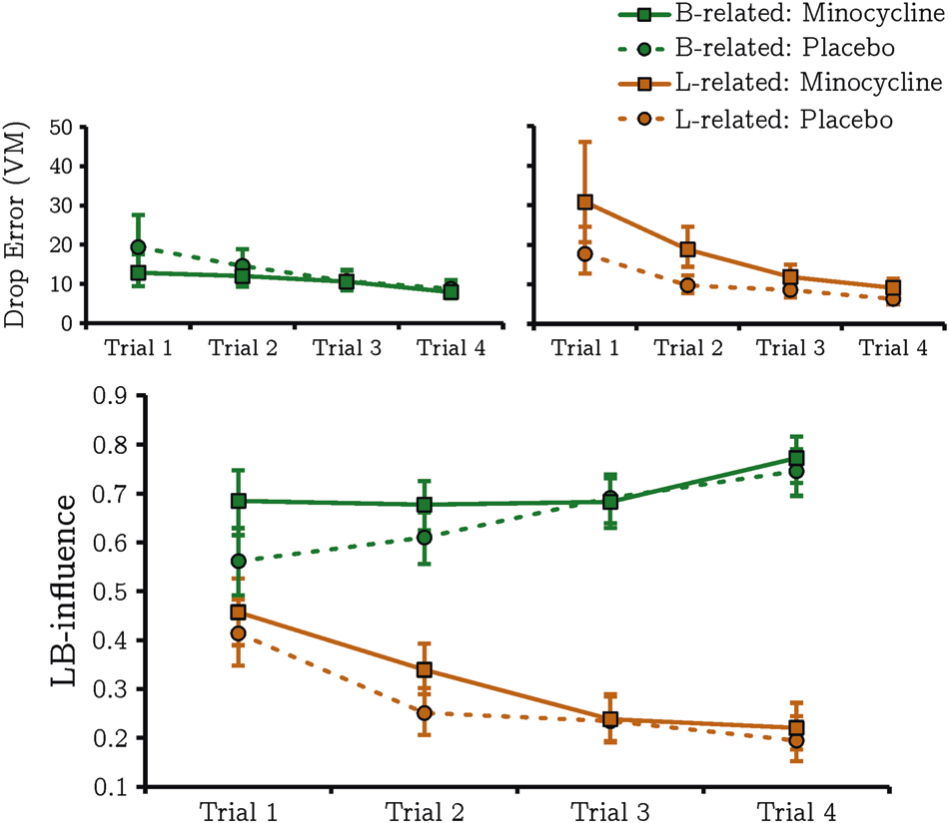
Behavioural performance on the virtual navigation task. Drop error statistics (**top row**) and LB-influence scores (**lower row**) broken down by drug, navigation condition and trial number. Error bars represent ±95% confidence intervals. VM = virtual metres. Significant difference in LB-influence scores on trial 1 are observed as this analysis only included trials after object locations were differentially trained.

Drop-error data (FEEDBACK) as a function of navigational condition, trial number and drug are displayed in Fig. 2 (top row). As anticipated, the GLMM identified a significant main effect of trial indicating that drop errors for both L- and B-related objects decreased as training progressed: *t*_980_ = 11.035, *p* < 0.001. The model also highlighted a significant trial by navigation condition interaction, *t*_980_ = 2.922, *p* = 0.004, with the reduction in drop errors occurring more rapidly for boundary- relative to landmark-following objects. In line with our prior prediction, we also observed a significant drug by navigation condition interaction, *t*_980_ = 5.264, *p* < 0.001. Although analysis of this interaction showed our predicted reduction in drop errors for boundary-related objects on minocycline, *t*_980_ = 1.972, *p* = 0.049, it also showed that minocycline increased drop errors for L-related objects, *t*_980_ = 6.374, *p* < 0.001. Furthermore, we also observed a significant main effect of drug with drop errors being significantly greater (i.e. worse performance overall) when on minocycline versus placebo, *t*_980_ = 3.295, *p* = 0.001.

We next examined the effects of minocycline on LB-influence (REPLACE) scores (Fig. 2, bottom row). Firstly, this identified a main effect of navigational condition confirming that LB-influence scores were significantly higher for B- compared to L-related objects, *t*_980_ = 23.940, *p* < 0.001. We also observed the expected navigation condition by trial interaction with LB-influence increasing for B- and decreasing for L-related objects as training progressed: *t*_980_ = 9.900, *p* < 0.001. However, the model further revealed a main effect of drug indicating that LB-influence scores were, in general, higher (more dependent on boundary) when on minocycline, *t*_980_ = 3.140, *p* = 0.002.

This suggests that minocycline enhances the use of boundary- compared to landmark-based navigational systems. Our drop-error findings suggest that this may marginally improve navigational performance for boundary-based objects, but this is outweighed by impaired landmark-based navigation resulting in an overall decrease in performance. In support of this, we observed no significant drug by navigation condition interaction on LB-influence: *t*_*980*_ = 0.070, *p* = .944 suggesting that minocycline boosts use of boundary- compared to landmark-based navigational systems, even in conditions where this adversely affects performance.

### Effects of BMI on behaviour

**Fig. 3:**
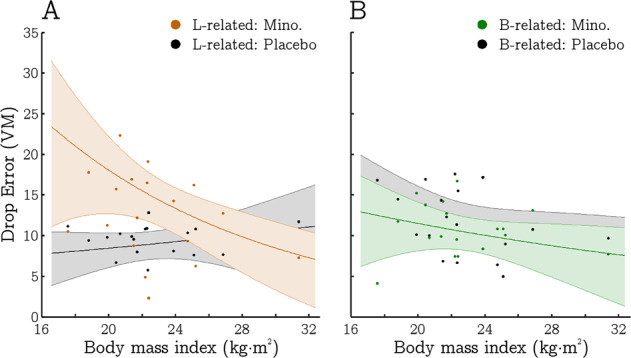
Relationship between body mass index (BMI) and drop errors (accuracy) in the virtual-navigation task plotted by experimental condition. **a** drop error scores from the landmark-related condition, **b** drop error scores from the boundary-related condition. Grey and coloured shadings relate to the placebo and minocycline sessions, respectively. Error bars indicate 95% confidence intervals.

Inclusion of BMI in the models identified two further significant effects of interest in the analysis of drop errors: A significant BMI by drug interaction (*t*_976_ = 4.276, *p* < 0.001) and a three-way interaction between BMI, drug, and navigation condition (*t*_976_ = 3.442, *p* < 0.001) that superseded this. This indicated that the degree to which landmark related navigation was adversely affected by minocycline was dependent on participant BMI, i.e. minocycline had a more detrimental effect when BMI was lower, and this effect was specific to the landmark-related condition (Fig. 3). No significant main effects of BMI, or a BMI by navigation condition interaction (*t*_976_’s < 1.096, *p*’s > 0.273) were observed, though the inclusion of BMI led to the beneficial effect of minocycline on boundary-related drop errors falling below the threshold of statistical significance, *t*_976_ = 1.689, *p* = 0.092.

### BOLD effects related to spatial navigation

We first examined whether landmark and boundary-based navigation were associated with differential levels of activity in our four ROIs. Here, we only looked for effects that were evident in the placebo condition so that the results were not influenced by any potential effects of minocycline. During the REPLACE phase (memory retrieval) we observed no significant main effects or interactions between navigation condition and LB-influence in any ROI (largest effect: *t*_982_ = 1.260, *p* = 0.208). Similarly, during the FEEDBACK phase (memory encoding) we observed no significant main effects or interactions between navigation condition and drop error (largest effect: *t*_973_ = 1.403, *p* = 0.161). As such, we failed to find any evidence of functional specialisation in our a priori ROIs.

In contrast, our whole-brain analyses did identify spatial memory effects of interest. Specifically, a number of regions exhibited a negative correlation between drop error and BOLD activity during the FEEDBACK phase of both landmark- and boundary-related trials (see Supplementary Fig. S2). This implies that more accurate responses were associated with higher levels of BOLD activity in both navigation conditions in these regions. Regions showing this effect included a large cluster in the lingual gyrus extending into the fusiform gyrus and posterior hippocampus (bilaterally). On the left side, this cluster also included a region of the retrosplenial cortex. Furthermore, bilateral effects were observed in the inferior parietal lobe, superior temporal gyrus, inferior frontal gyrus, and anterior cingulate cortex. No other effects of interest were detected in the placebo session during either the REPLACE or FEEDBACK phases.

### Effects of minocycline

**Fig. 4:**
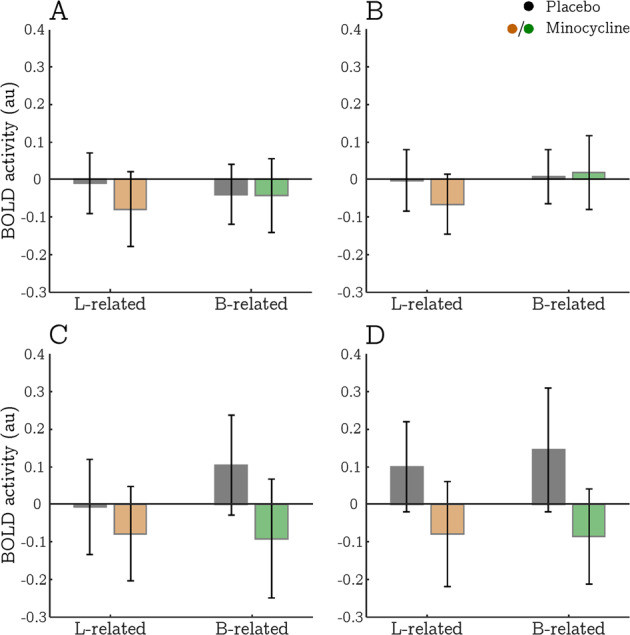
Mean estimates of BOLD activity during FEEDBACK (memory encoding) in all a priori ROIs plotted by experimental condition: navigation type (landmark vs boundary) and drug (placebo vs minocycline). Each panel corresponds to a different ROI—**a** left hippocampus; **b** right hippocampus; **c** left caudate; **d** right caudate. Grey and coloured shadings relate to the placebo and minocycline sessions, respectively. Error bars indicate 95% confidence intervals.

The ROI analyses revealed that minocycline was associated with a significant reduction in right caudate activity during memory encoding (FEEDBACK) regardless of navigational condition (main effect of minocycline: *t*_977_ = 2.992, *p* = 0.003; Fig. 4). A similar though non-significant effect was also observed in the left caudate (*t*_973_ = 1.924, *p* = 0.053). The ROI analyses found no other main effects or interactions involving minocycline during either the REPLACE or FEEDBACK phases.

To test whether minocycline exerted a statistically larger effect in the caudate than the hippocampus ROIs, we compared the main effect of minocycline during FEEDBACK across regions within the same hemisphere using an unequal variances *t*-test (residual degrees of freedom were approximated with the Welch-Satterthwaite equation [33]). These comparisons confirmed a larger effect in the right, but not the left caudate; *t*_1492_ = 2.288, *p* = 0.022 and *t*_1672_ = 1.179, *p* = 0.239, respectively. Taken together, these results demonstrate that right caudate activity during both landmark- and boundary-based navigation, was attenuated under minocycline.

The whole-brain analysis also revealed significant main effects of minocycline during the FEEDBACK phase in five other cortical regions (see Supplementary Fig. S3). These were located in the visual cortex bilaterally (V1–V3), the right inferior parietal lobe, and right middle frontal gyrus. As above, each of these effects indicated that minocycline was associated with a significant reduction in BOLD activity during memory encoding regardless of navigational condition. No other main effects or interactions involving minocycline, including any interactions with BMI, were detected across analyses.

## Discussion

Here we show that minocycline differentially modulated memory performance: moderately reducing errors during boundary-based navigation yet increasing landmark-based replacement errors. Related to this, minocycline appeared to enhance participants’ reliance on boundary- compared to landmark-based learning systems during navigation. These findings are broadly in line with our predictions that minocycline would modulate hippocampal-mediated memory performance which is thought to underpin boundary-based navigation. Importantly, however, the boost in performance for boundary-based navigation was only modest, while the impairment of landmark-based navigation was substantial, and together these effects resulted in an overall impairment in spatial memory performance. Furthermore, the moderate reduction in boundary-related errors was transient, being evident only at the early stages of learning when boundary-related information is known to take precedence [34, 35]. Minocycline-induced impairments in landmark-based navigation were also more pronounced in participants with low or normal BMI (i.e. <25).

Our imaging data are consistent with the interpretation that minocycline exerted more of a negative effect on landmark-based navigation than a positive effect on boundary-based navigation; minocycline was associated with a significant reduction in BOLD activity in the right striatum. This was evident when participants received error-related feedback. Although we did not find the predicted relationship between striatal activity and behavioural performance, previous research has heavily implicated the striatum in guiding learning by processing prediction error signals [36]. Furthermore, this mechanism is thought to be particularly relevant to landmark-related navigation [37]. Aside from this reduction in striatal activity, we also identified a number of similar effects in five other cortical regions including the visual cortex (V1–V3), inferior parietal lobe, and middle frontal gyrus where minocycline was associated with a significant reduction in BOLD activity during memory encoding regardless of navigational condition.

Importantly, the effects of minocycline on both behavioural performance and BOLD activity were evident regardless of whether landmark- or boundary-related information was being processed. Thus, although landmark-based navigation may be particularly dependent on striatal function, our results suggest that this region also influences boundary-based navigation. In line with this, we found no evidence that landmark- or boundary-based navigation differentially activated either the hippocampus or the striatum, thought the posterior hippocampus did exhibit BOLD effects that tracked trial-by-trial retrieval success. This contrasts with findings from a previous fMRI study which reported that boundary-based navigation preferentially activates the hippocampus, and landmark-based navigation the striatum [27]. The reasons for this discrepancy are not clear, however, it is notable that a number of other studies have suggested that there is no strict functional dichotomy between the hippocampal and striatal nuclei during navigation [38–40].

Our results are consistent with those from the rodent literature which have reported moderate benefits of minocycline on hippocampally-mediated memory processes, though only in either aged animals [21] or rodent models of Alzheimer’s [20]. In addition, while we did not explicitly predict the negative effect of minocycline on landmark-based navigation and striatal function, these results are perhaps unsurprising given that microglia play multiple roles in supporting healthy cognition by coordinating a wide array of fundamental processes linked to learning and memory [4]. Furthermore, the antimicrobial effects of minocycline are likely to disrupt the gut microbiota which indirectly influences memory performance by modulating LTP and neurogenesis [41, 42].

Another notable finding was that the detrimental effects of minocycline on behaviour were negatively related to BMI, with more detrimental effects observed in low or normal weight (BMI < 25) individuals. This may simply reflect a dose-dependent relationship since the minocycline dose was not adjusted by participant mass. However, it may also relate to the differential effects of inhibiting microglial activation reported in rodents where both microglial and microbiota functions are known to depend on metabolic status [43, 44]. In particular, while microglia play important roles in sustaining cognitive performance in health, heightened microglial activity in obesity has been linked to associated impairments in cognition [43, 44]. Given this, the inhibitory effects of minocycline on microglial activation may actually aid cognition in obesity. In line with this, though minocycline was associated with poorer memory performance in our sample of healthy individuals, we note that the one individual with a BMI in the obese range (>30) actually showed a modest improvement in memory performance on minocycline. As such, our results hint that minocycline may have beneficial effects on some aspects of cognition for this group, though this will clearly need to be explicitly addressed in future studies.

It is notable that studies investigating the role of neuroinflammatory processes, microglia and effects of minocycline in rodent learning have focussed almost exclusively on the medial temporal lobes, particularly the hippocampus [4]. Within the rodent hippocampus, microglia are thought to affect cognition via modulation of LTP and neurogenesis [11, 16, 45, 46]. Our findings suggest that minocycline also likely affects neuronal function in other brain regions including striatum, which in normal-weight individuals is associated with negative effects on behaviour. Whether this results from direct effects of minocycline on neuronal populations within these regions or from modulation in functionally connected systems cannot be determined here. However, irrespective of the neural mechanisms, the current findings suggest that minocycline is unlikely to confer any therapeutic benefit in treating or preventing generalised cognitive decline involving neuroinflammatory processes and adds to a growing body of evidence that minocycline offers negligible neuroprotective effects [25, 47, 48].

In summary, we show that minocycline significantly modulates learning and memory processes in healthy human participants. Overall, this had the effect of decreasing memory performance, particularly when learning spatial locations relative to landmarks. This effect was most pronounced in participants with a low or normal BMI (i.e. <25). Together, these observations lend additional support to theories implicating microglia in learning and memory functions in humans. Future studies probing the effects of minocycline on other cognitive processes will be required to support our findings and, in particular, investigate the potential for any beneficial effect of minocycline in obesity. Intracranial electrode recordings would allow a direct examination of minocycline’s effects of neuronal firing rates and synaptic potentiation in different brain regions. Furthermore, Translocator (TSPO) positron emission tomography [49–51] could direct investigate whether the cognitive effects of minocycline directly correspond to microglial activity.

## Funding and disclosure

This article was funded by a Wellcome Trust Fellowship awarded to NAH (Grant Number: 093881/Z/10/Z). SCB was supported by an Economic and Social Research Council studentship (ES/J500173/1). In addition, CMB and SCB are funded by a European Research Council Consolidator Grant awarded to CMB (Project: EVENTS).

## Supplementary information

Supplementary Information

Supplementary Figure 1

Supplementary Figure 2.

Supplementary Figure 3.

## Supplementary information

**Supplementary Information** accompanies this paper at (10.1038/s41386-020-00811-8). Anonymised behavioural data, neuroimaging data, and analysis scripts are freely available on the Open Science Framework (https://osf.io/kdb49/).
