# Supplementary material for: Minocycline differentially modulates human spatial memory systems
Source: Neuropsychopharmacology. 2020 Aug 24;45(13):2162–9. doi: 10.1038/s41386-020-00811-8 (PMC7784680; doi:10.1038/s41386-020-00811-8)
Supplement: Supplementary file 1 — Supplementary Information [file 41386_2020_811_MOESM1_ESM.docx]

Supplementary Information for:
Minocycline differentially modulates human spatial memory systems

Sam C Berens, Chris M Bird, Neil A Harrison

# Supplementary Figures


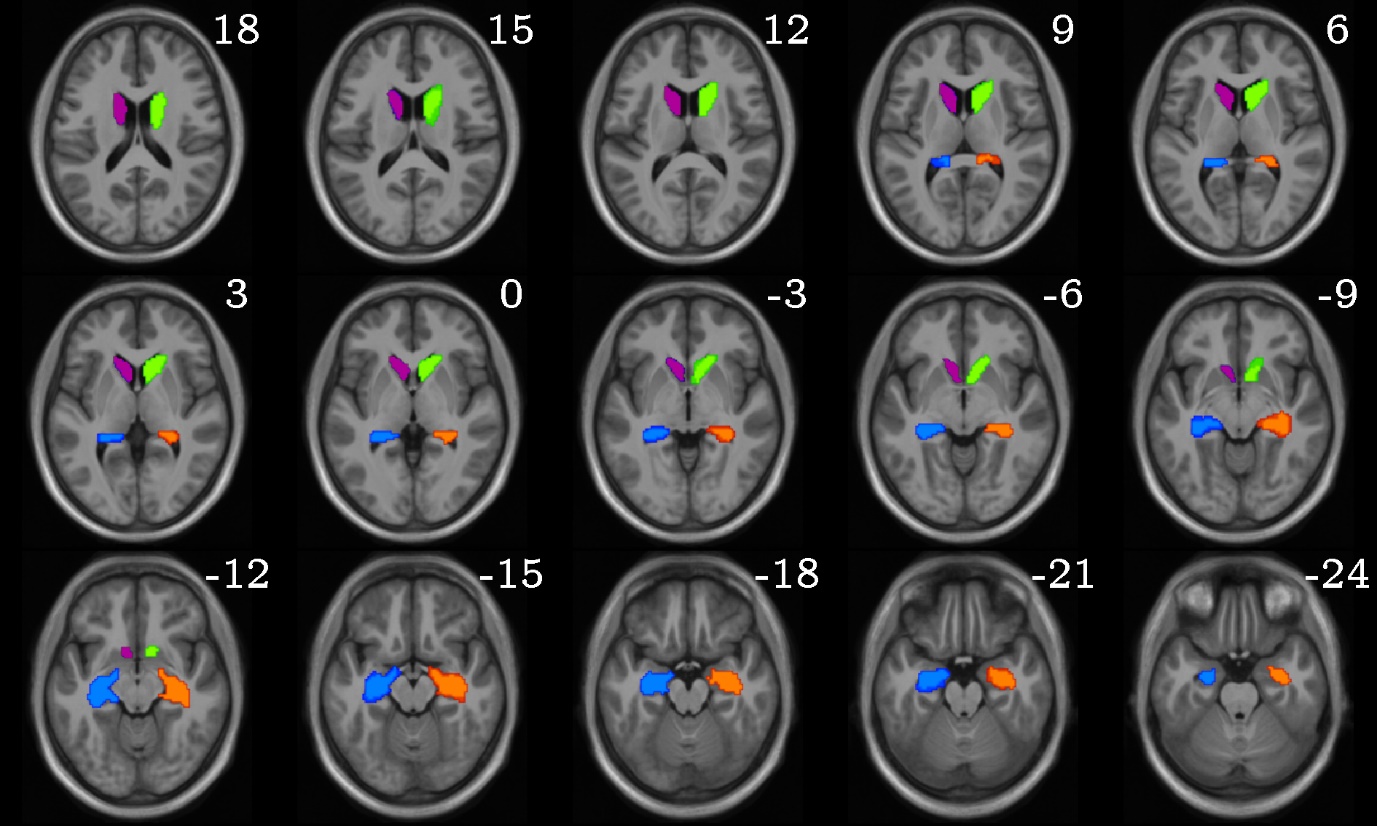


***Supplementary Figure 1.*** *Regions of interest used the a priori analyses, specifically the left and right caudate nucleus, and the left and right hippocampus. All masks defined from the Automated Anatomical Labelling (AAL) atlas (Tzourio-Mazoyer et al., 2002). The anatomical underlay is a mean image of normalised MP-RAGE scans from all participants included in the analysis. The colour map indicates t-values for the statistical contrast and numerals printed north east of each sub-plot denote the z co-ordinate in MNI space.*


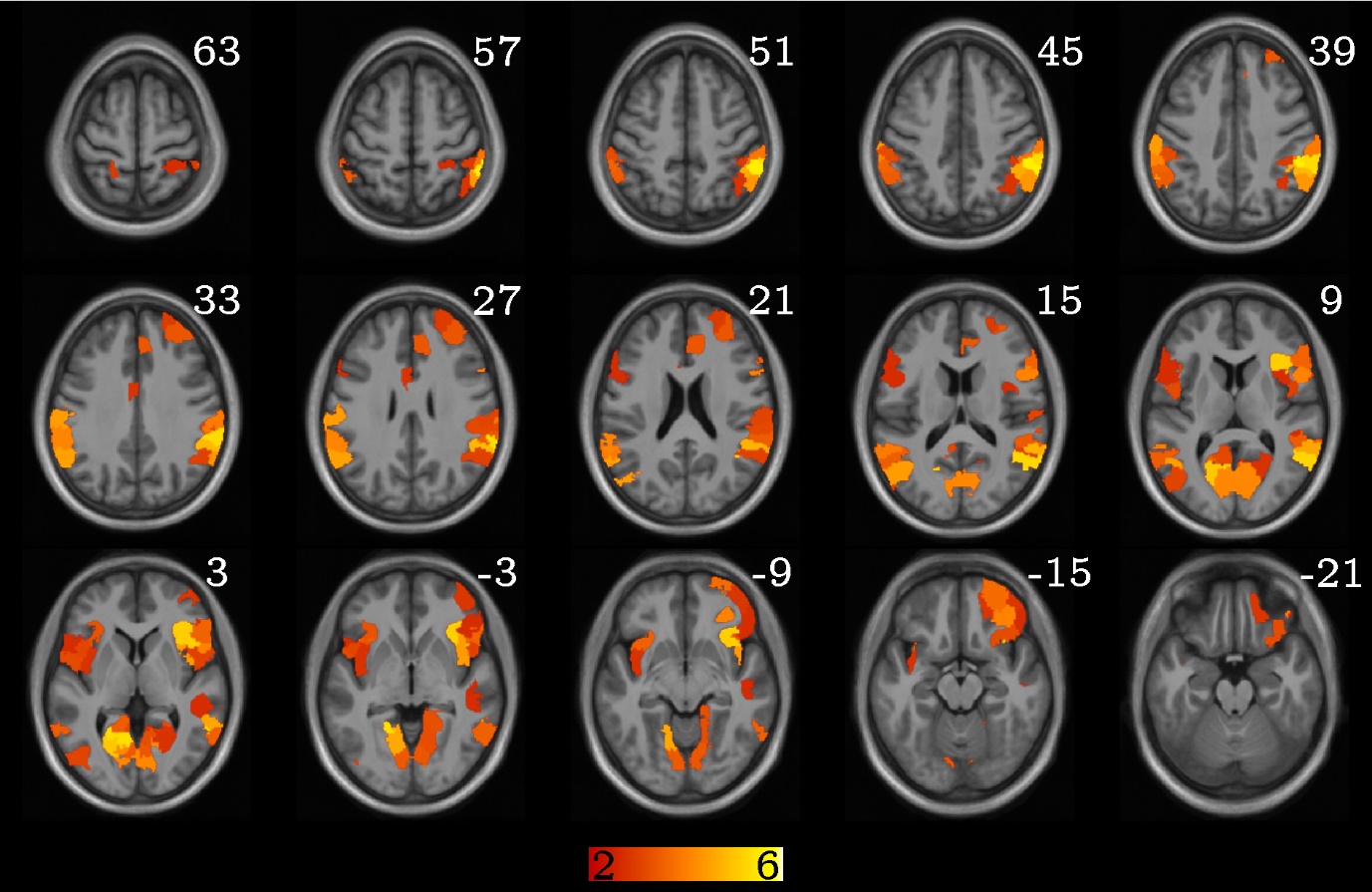


***Supplementary Figure 2.*** *Regions that exhibited a* *negative correlation between drop error and BOLD activity during FEEDBACK in the spatial navigation task. In each of these regions, more accurate responses were associated with higher levels of BOLD activity across both landmark- and boundary-related trials. These effects were identified in the whole-brain analysis and are statistically significant after correcting for false discovery rate across 400 functionally defined brain parcels (see Methods). The anatomical underlay is a mean image of normalised MP-RAGE scans from all participants included in the analysis. The colour map indicates t-values for the statistical contrast and numerals printed north east of each sub-plot denote the z co-ordinate in MNI space.*


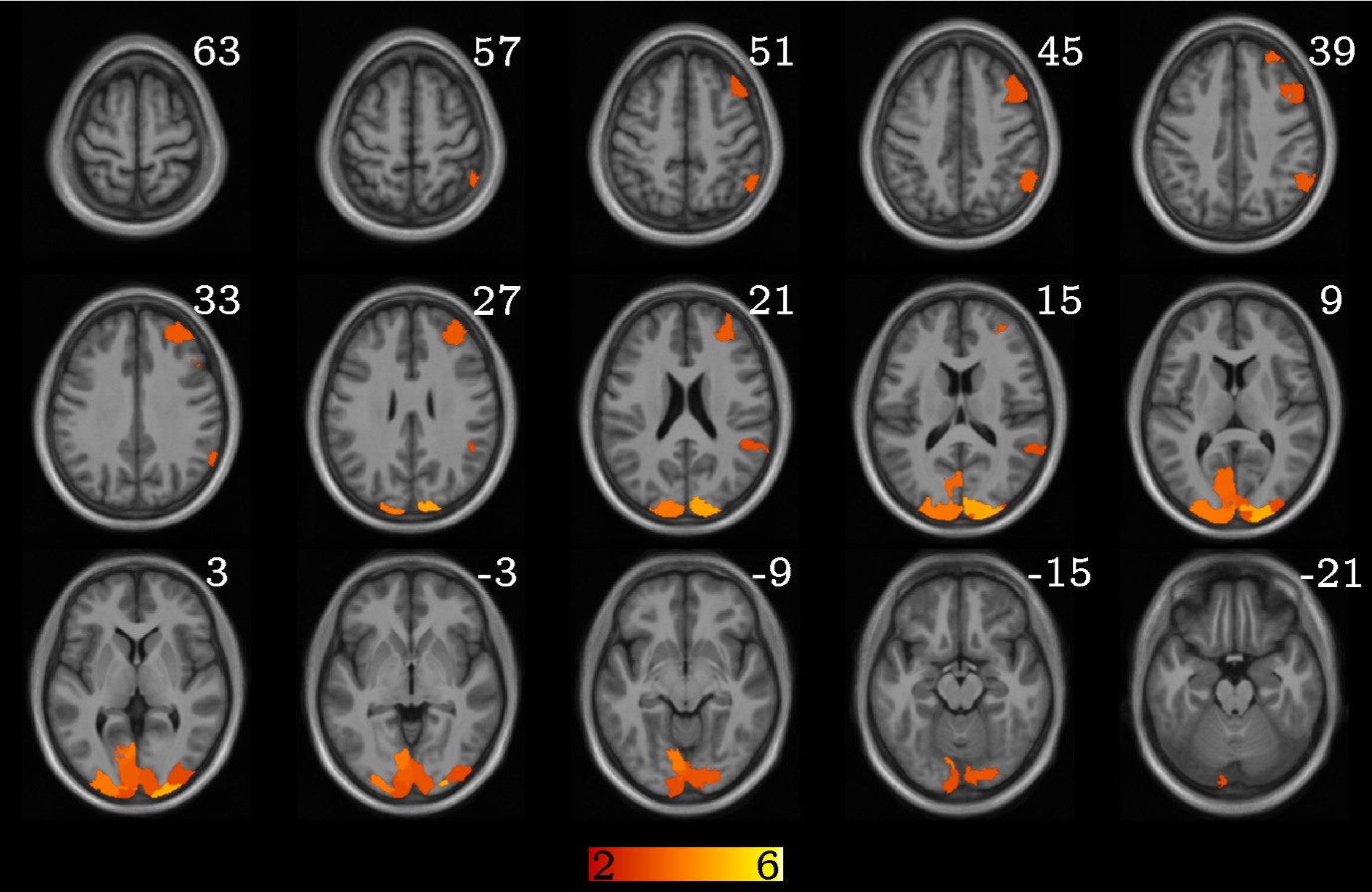


***Supplementary Figure 3.*** *Regions that yielded a main effect of minocycline during FEEDBACK in the spatial navigation task. All of these areas produced lower levels of BOLD activity while on minocycline (averaged across both landmark- and boundary-related conditions). These effects were identified in the whole-brain analysis and are statistically significant after correcting for false discovery rate across 400 functionally defined brain parcels (see Methods). The anatomical underlay is a mean image of normalised MP-RAGE scans from all participants included in the analysis. The colour map indicates t-values for the statistical contrast and numerals printed north east of each sub-plot denote the z co-ordinate in MNI space.*

# Supplementary Methods

**MRI data acquisition and Pre-processing:** T2*-weighted echo planar images (EPIs) were acquired in a 1.5 Tesla Siemens Avanto scanner, equipped with a 32-channel phased-array head coil using a -30° tilted acquisition to reduce orbitofrontal dropout. Each volume provided whole brain coverage (34 interleaved ascending 3mm slices, 0.6mm inter-slice gap, echo-time 43msec: TR 2.52s). A high-resolution magnetization-prepared-rapid-acquisition-gradient-echo (MPRAGE) T1-weighted structural scan was acquired to aid group level anatomical localization.

Image pre-processing was performed in SPM8 ([www.fil.ion.ucl.ac.uk/spm](http://www.fil.ion.ucl.ac.uk/spm)). EPI volumes were spatially realigned to the first image in the time series, realigned to their respective MPRAGE image, warped to MNI space using transformation parameters derived from normalization of the MPRAGE scans (DARTEL toolbox) then spatially smoothed with an isotropic 8mm FWHM Gaussian kernel.

Prior to statistical analysis, the EPI data were decorrelated with two nuisance regressors: *1)* a simple linear trend, and *2)* the mean signal intensity from all white matter voxels. This decorrelation step was needed in order to remove nuisance low frequency scanner drift signals from the EPI data. In addition, all first level models of the EPI data included a Fourier basis set implementing a 1/128 Hz high-pass filter. Given that spatial learning is correlated with time, not removing these low-frequency components was found to result in erroneous BOLD effects.

**Statistical validation and inference:** To ensure that each mixed-effect regression model was not unduly influenced by outlying data points, we systematically excluded observations that produced unexpectedly large residuals (more than 3 standard deviations above or below model estimates). This was conducted regardless of condition and so did not bias the analyses to finding an effect. Further, a highly similar pattern of results was seen when including outliers, supporting the robustness of our findings. All *p*-vales are reported as two-tailed statistics. Unless otherwise stated, we only report effects surviving Bonferroni correction across our 4 *a priori* regions of interest. For our supplementary analysis testing for BOLD effects in the 400 brain parcels, we only report results that survive Benjamini–Hochberg false discovery rate (FDR) correction [S1].

# Supplementary References

[S1] Benjamini Y, Hochberg Y. Controlling the False Discovery Rate: A Practical and Powerful Approach to Multiple Testing. J R Stat Soc Ser B. 1995;57:289–300.
