# Supplementary figures and images for: Minocycline differentially modulates human spatial memory systems
Source: Neuropsychopharmacology. 2020 Aug 24;45(13):2162–9. doi: 10.1038/s41386-020-00811-8 (PMC7784680; doi:10.1038/s41386-020-00811-8)

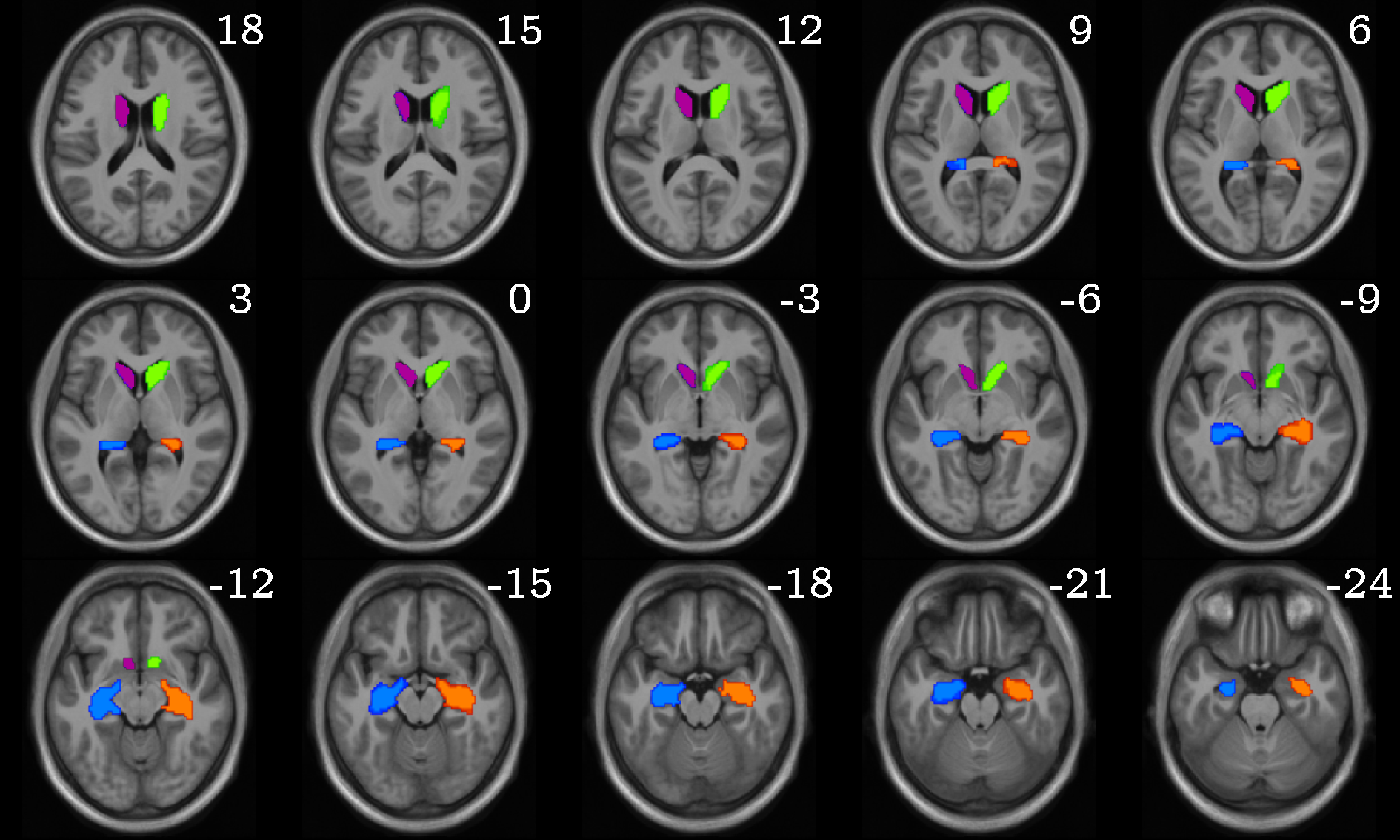

Supplement: Supplementary file 2 — Supplementary Figure 1 [file 41386_2020_811_MOESM2_ESM.tif]

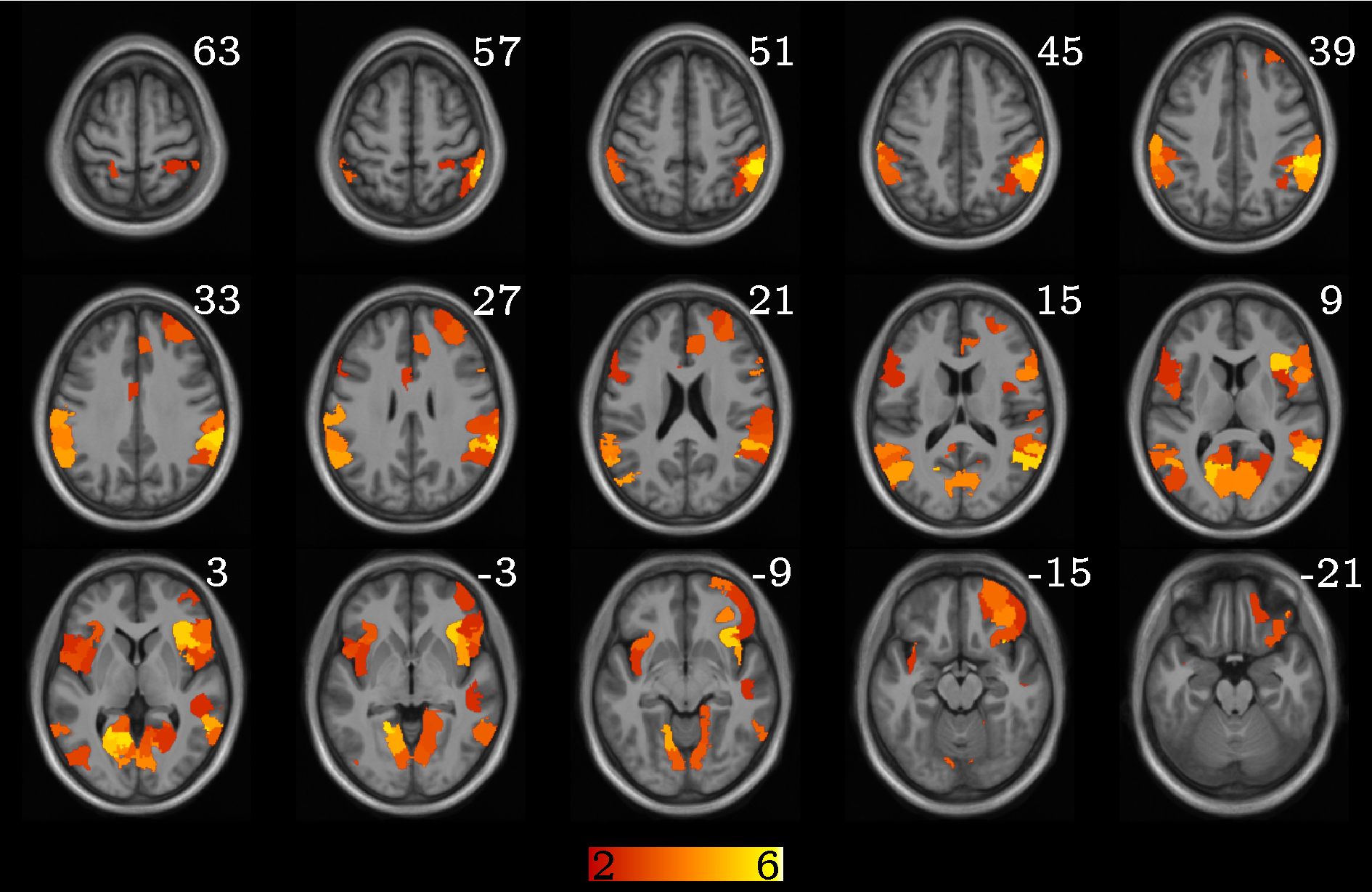

Supplement: Supplementary file 3 — Supplementary Figure 2. [file 41386_2020_811_MOESM3_ESM.tif]

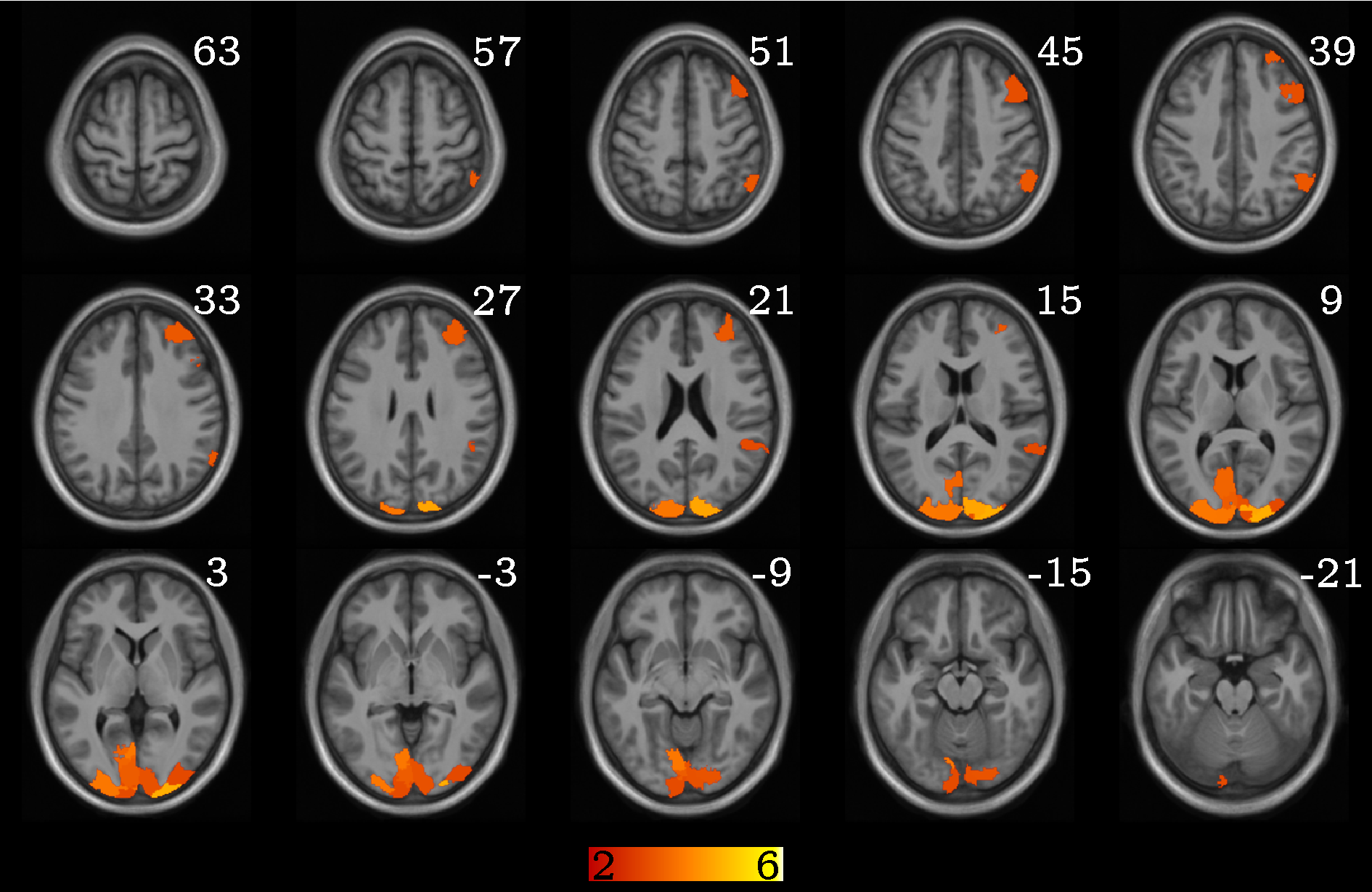

Supplement: Supplementary file 4 — Supplementary Figure 3. [file 41386_2020_811_MOESM4_ESM.tif]
